# Supplementary material for: Antioxidant and Anti-α-Glucosidase Activities of Various Solvent Extracts and Major Bioactive Components from the Fruits of Crataegus pinnatifida
Source: Antioxidants (Basel). 2022 Feb 6;11(2):320. doi: 10.3390/antiox11020320 (PMC8868160; doi:10.3390/antiox11020320)
Supplement: Supplementary file 1 [file antioxidants-11-00320-s001.zip › antioxidants-1544449-supplementary.pdf]

Supplementary data

Antioxidant and Anti-α-glucosidase Activities of Various Solvent Extracts and Major Bioactive Components from the Fruits of *Crataegus pinnatifida*

Yen-Ting Lin [1], Hsiang-Ru Lin [2], Chang-Syun Yang [1], Chia-Ching Liaw [3,4], Ping-Jyun Sung [5], Yueh-Hsiung Kuo [6,7,8], Ming-Jen Cheng [9,*] and Jih-Jung Chen [1,6,*]

[1] Department of Pharmacy, School of Pharmaceutical Sciences, National Yang Ming Chiao Tung University, Taipei 112, Taiwan; ytl97.ps09@nycu.edu.tw (Y.-T.L.); tim0619@nycu.edu.tw (C.-S.Y.)

[2] Department of Chemistry, College of Science, National Kaohsiung Normal University, Kaohsiung 824, Taiwan; t3136@nknu.edu.tw (H.-R.L.)

[3] National Research Institute of Chinese Medicine, Ministry of Health and Welfare, Taipei 112, Taiwan; liawcc@nricm.edu.tw (C.-C.L.)

[4] Department of Biochemical Science and Technology, National Chiayi University, Chiayi 600, Taiwan

[5] National Museum of Marine Biology and Aquarium, Pingtung 944, Taiwan; pjsung@nmmba.gov.tw

[6] Department of Medical Research, China Medical University Hospital, China Medical University, Taichung 404, Taiwan; kuoyh@mail.cmu.edu.tw (Y.-H.K.)

[7] Department of Biotechnology, Asia University, Taichung 413, Taiwan

[8] Department of Chinese Pharmaceutical Sciences and Chinese Medicine Resources, College of Pharmacy, China Medical University, Taichung 404, Taiwan

[9] Bioresource Collection and Research Center (BCRC), Food Industry Research and Development Institute (FIRDI), Hsinchu 300, Taiwan

* Correspondence: jjungchen@nycu.edu.tw (J.-J.C.); chengfirdi@gmail.com (M.-J.C.); Tel.: +886-2-2826-7195 (J.-J.C.); Fax: +886-2-2823-2940 (J.-J.C.)

Contents

Table S1. Retention time, LODs, LOQs, and regression analysis for three components in *Crataegus pinnatifida* in normal- and reverse phase.. S3

Figure S1. The TLC graphs of isolated compounds…..S3

Figure S2. Reverse-phase HPLC chromatogram of isolated pure compounds........................ S3

Figure S3. Reverse-phase HPLC chromatogram of methanol extract.......................................S4

Figure S4. Reverse-phase HPLC chromatogram of ethanol extract.. S4

Figure S5. Reverse-phase HPLC chromatogram of acetone extract.. S4

Figure S6. Reverse-phase HPLC chromatogram of ethyl acetate extract.................................. S5

Figure S7. Reverse-phase HPLC chromatogram of dichloromethane extract.......................... S5

Figure S8. Reverse-phase HPLC chromatogram of chloroform extract…...................................S5

Figure S9. Reverse-phase HPLC chromatogram of *n*-hexane extract……................................. S6

Table S1. Retention time, LODs, LOQs, and regression analysis for four components in *Crataegus pinnatifida* in reverse-phase.

Compounds	T_m (min) [a]	Regression equation	Correlation coefficient	LOD (μg/mL) [a]	LOQ (μg/mL) [a]
Epicatechin	4.06	y = 32979x+5882.9	0.9999	0.20	0.67
Chlorogenic acid	37.37	y = 45096x-3594.4	0.9999	0.15	0.50
Hyperoside	47.12	y = 97065x+4134.8	0.9998	0.10	0.33
Procyanidin B2	101.20	y = 101724x+6159.5	0.9997	0.12	0.40

[a] T_m: Retention time; LOD: Limit of detection ; LOQ : Limit of quantification

n-Hexane/Acetone = 2:1 CH_2Cl_2/MeOH = 5:1 CH_2Cl_2 /MeOH = 5:1   *n*-Hexane/Acetone = 2:1

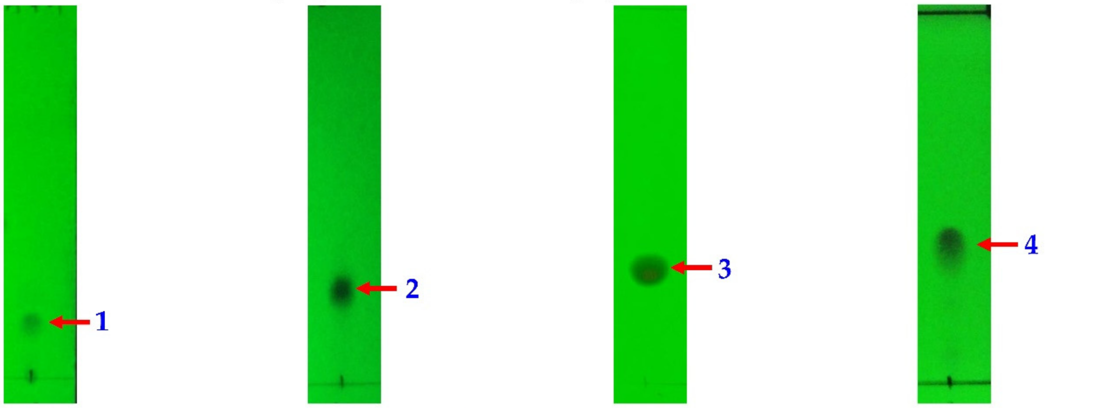

Epicatechin (1) (R_f = 0.12)   Chlorogenic acid (2) (R_f = 0.23)   Hyperoside (3) (R_f = 0.30)   Procyanidin B2 (4) (R_f = 0.35)

Figure S1. The TLC graphs of isolated compounds.

(A) Epicatechin (500 μL), 280 nm   (B) Chlorogenic acid (500 μL), 280 nm

(C) Hyperoside (500 μL), 280 nm   (D) Procyanidin B2 (500 μL), 280 nm

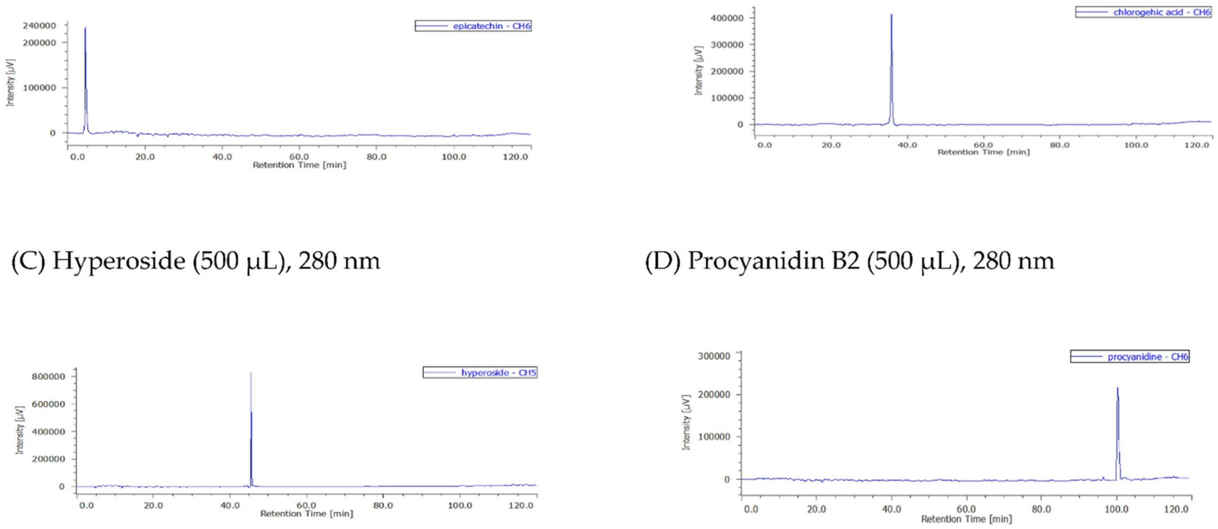

Figure S2. Reverse-phase HPLC chromatogram of isolated pure compounds (A to D).

Methanol (500 µL), 280 nm

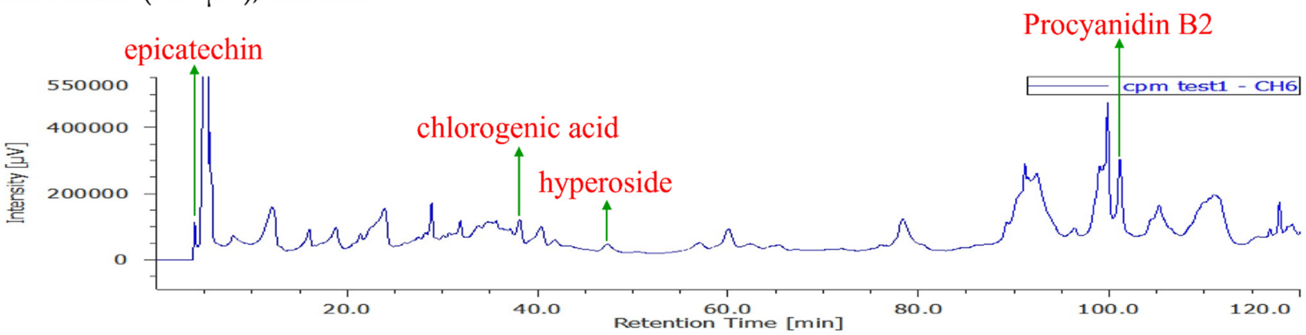

Figure S3. Reverse-phase HPLC chromatogram of methanol extract.

Ethanol (500 µL), 280 nm

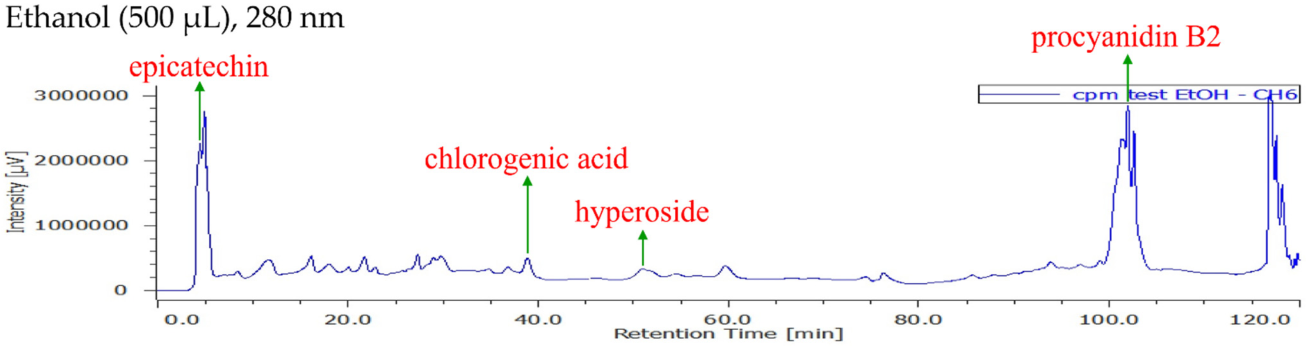

Figure S4. Reverse-phase HPLC chromatogram of ethanol extract.

Acetone (500 µL), 280 nm

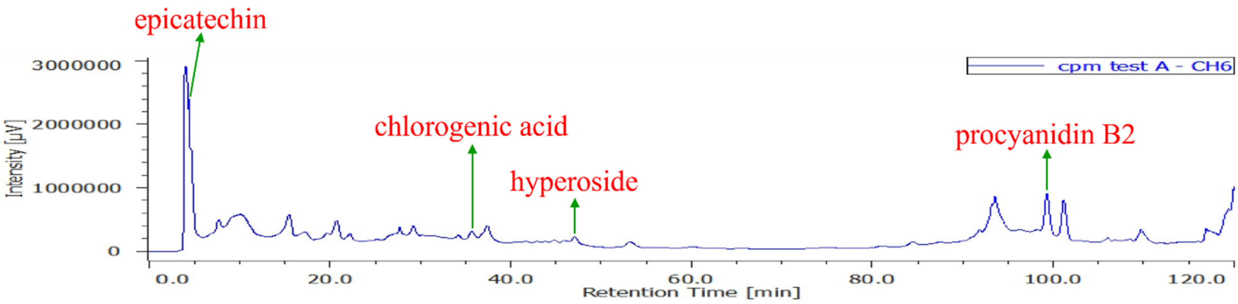

Figure S5. Reverse-phase HPLC chromatogram of acetone extract.

Ethyl acetate (500 µL), 280 nm

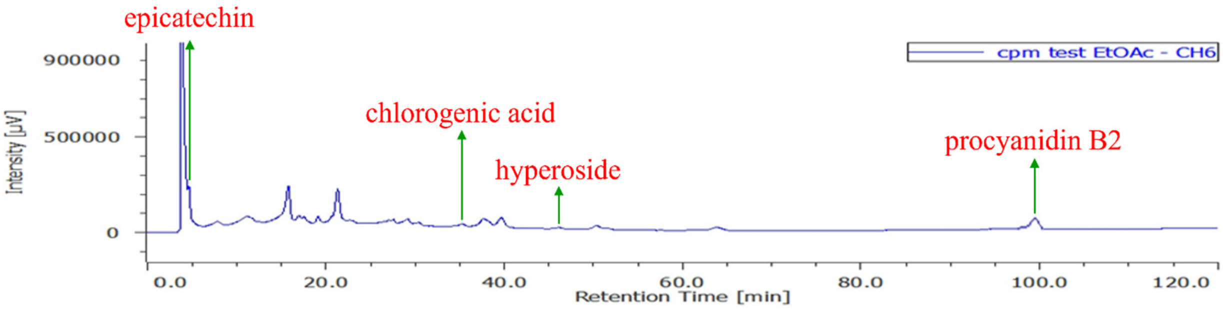

Figure S6. Reverse-phase HPLC chromatogram of ethyl acetate extract.

Dichloromethane (500 µL), 280 nm

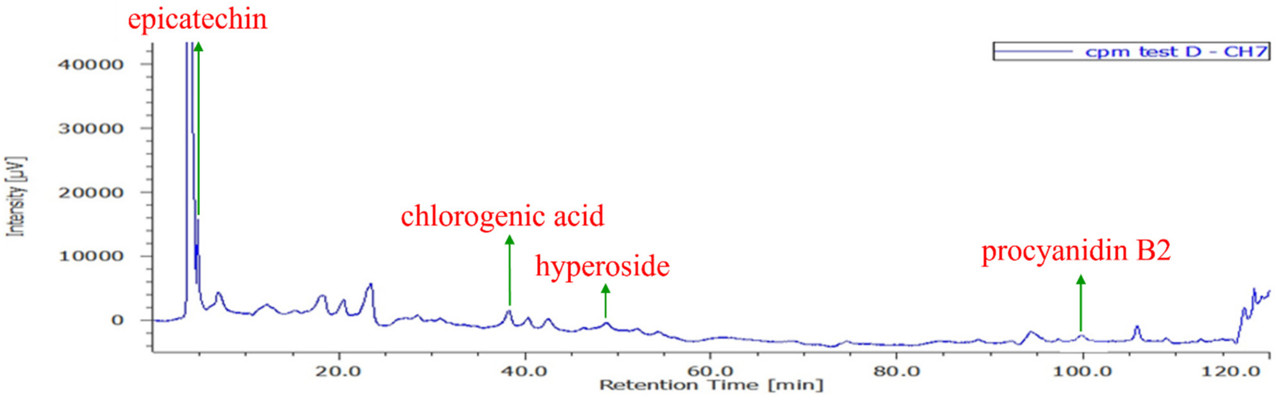

Figure S7. Reverse-phase HPLC chromatogram of dichloromethane extract.

Chloroform (500 µL), 280 nm

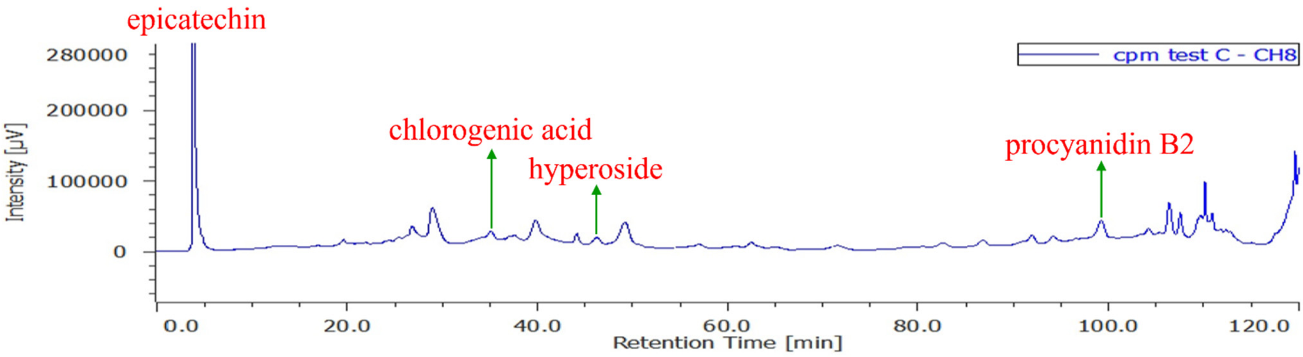

Figure S8. Reverse-phase HPLC chromatogram of chloroform extract.

n-Hexane (500 μL), 280 nm

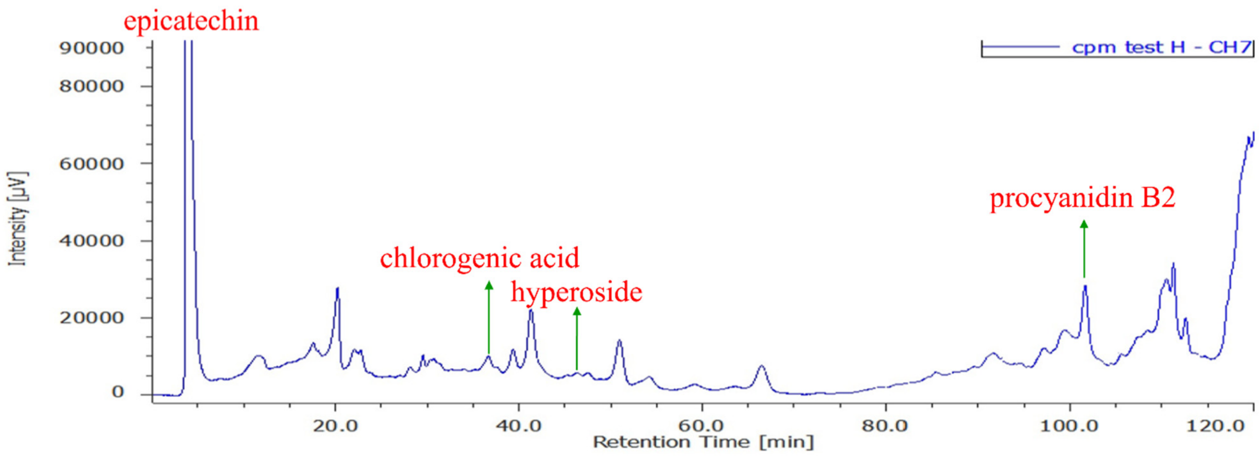

Figure S9. Reverse-phase HPLC chromatogram of *n*-hexane extract.
